# Automated Online Flow Cytometry Advances Microalgal Ecosystem Management as *in situ*, High-Temporal Resolution Monitoring Tool

**DOI:** 10.3389/fbioe.2021.642671

**Published:** 2021-03-23

**Authors:** Iris Haberkorn, Cosima L. Off, Michael D. Besmer, Leandro Buchmann, Alexander Mathys

**Affiliations:** ^1^Sustainable Food Processing Laboratory, Institute of Food, Nutrition and Health, ETH Zurich, Zurich, Switzerland; ^2^onCyt Microbiology AG, Zurich, Switzerland; ^3^Bühler AG, Uzwil, Switzerland

**Keywords:** *Chlorella vulgaris*, phenotypic fingerprinting, online flow cytometry, microalgae, prokaryotes

## Abstract

Microalgae are emerging as a next-generation biotechnological production system in the pharmaceutical, biofuel, and food domain. The economization of microalgal biorefineries remains a main target, where culture contamination and prokaryotic upsurge are main bottlenecks to impair culture stability, reproducibility, and consequently productivity. Automated online flow cytometry (FCM) is gaining momentum as bioprocess optimization tool, as it allows for spatial and temporal landscaping, real-time investigations of rapid microbial processes, and the assessment of intrinsic cell features. So far, automated online FCM has not been applied to microalgal ecosystems but poses a powerful technology for improving the feasibility of microalgal feedstock production through *in situ*, real-time, high-temporal resolution monitoring. The study lays the foundations for an application of automated online FCM implying far-reaching applications to impel and facilitate the implementation of innovations targeting at microalgal bioprocesses optimization. It shows that emissions collected on the FL1/FL3 fluorescent channels, harnessing nucleic acid staining and chlorophyll autofluorescence, enable a simultaneous assessment (quantitative and diversity-related) of prokaryotes and industrially relevant phototrophic *Chlorella vulgaris* in mixed ecosystems of different complexity over a broad concentration range (2.2–1,002.4 cells ⋅μL^–1^). Automated online FCM combined with data analysis relying on phenotypic fingerprinting poses a powerful tool for quantitative and diversity-related population dynamics monitoring. Quantitative data assessment showed that prokaryotic growth phases in engineered and natural ecosystems were characterized by different growth speeds and distinct peaks. Diversity-related population monitoring based on phenotypic fingerprinting indicated that prokaryotic upsurge in mixed cultures was governed by the dominance of single prokaryotic species. Automated online FCM is a powerful tool for microalgal bioprocess optimization owing to its adaptability to myriad phenotypic assays and its compatibility with various cultivation systems. This allows advancing bioprocesses associated with both microalgal biomass and compound production. Hence, automated online FCM poses a viable tool with applications across multiple domains within the biobased sector relying on single cell–based value chains.

## Introduction

Cellular agriculture and along with it renewable biobased materials relying on single-cell biorefineries as, for instance, those associated with yeasts, bacteria, and microalgae, are gaining momentum. Microalgae have attracted attention as a sustainable means of a next-generation biotechnological production system for the food, feed, pharmaceutical, nutraceutical, and biofuels sector. They are of emerging interest owing the sustainable notion of their connected value chains. Microalgal biomass is characterized by a beneficial composition with protein contents of up to 65%, depending on the species employed or lipid contents with a beneficial ratio of ω6- to ω3-polyunsaturated fatty acids. Industrial products extracted from microalgae, for instance, include β-carotene, lipids, polysaccharides, and vitamins such as vitamin B_12_, proteins, or phycocyanin (Hyka et al., 2013; Caporgno and Mathys, 2018; Canelli et al., 2020; Rischer et al., 2020).

The economization of microalgal bioprocesses remains a main target, which comprises optimizing the productivity and reproducibility of microalgal biomass and compound production. Flow cytometry (FCM) poses a viable technology for improving the feasibility of the bioprocesses associated with microalgal biorefineries. Microalgae are a diverse group of microorganisms differing in their morphology, ecology, physiology, and biochemistry. FCM enables a rapid and accurate discrimination and quantification of different cells, as well as a depiction of physiological states based on their inherent cell characteristics. So far, FCM has been applied for the monitoring of bioprocesses associated with, for example, astaxanthin, oil, or glucose production (Hyka et al., 2013). The development of automated tools adjunctive to FCM that enable online and inline culture monitoring further perpetuates the application of the technology for single-cell bioprocess management. Automated online FCM enables spatial and temporal landscaping, as well as investigations of rapid processes on a quantitative and phenotype-related base harnessing intrinsic cell features *in situ*, at real-time, and at high-temporal resolution.

An important aspect in optimizing the feasibility of microalgal bioprocesses in terms of reproducibility and productivity is associated with the management of culture ecologies. Phototrophic microalgae production as monocultures is not a realistic scenario on industrial scale. Additionally, culture contamination, for example, caused by the extraneous invasion of parasitic prokaryotic species or microalgal grazers, was reported as a primary bottleneck to impairing microalgal productivities. Culture contaminations can lead to biomass and consequently economic losses (Enzing et al., 2014). A real-time detection and quantification tool allows taking immediate countermeasures as a response to microbial disturbances caused by such contaminants or to the upsurge of prokaryotic counts during culture. Thus, it could contribute to the stability, reproducibility, and consequently productivity of microalgal feedstock production. FCM is advantageous over traditional techniques, such as plating, which are often laborious and fail to reflect complex ecosystems, as it allows for a fast and reproducible detection and enumeration of cultivable and non-cultivable microorganisms (Hammes and Egli, 2005). Microalgae can be easily distinguished from prokaryotic organisms or abiotic particles based on their size and granularity, i.e., forward (FSC) and sideward (SSC) scattered light intensities, respectively (Haberkorn et al., 2019). The nucleic acid content or pigment autofluorescence provide additional, distinctive features (Hammes and Egli, 2010; Hyka et al., 2013; Prest et al., 2013; Besmer et al., 2014).

These phenotypic properties reflecting inherent cellular features allow establishing FCM data analysis approaches that enable a community characterization beyond a detection and purely quantitative assessment. Props et al. (2016) established a data analysis approach relying on phenotypic fingerprinting that enabled the assessment of prokaryotic community dynamics in aquatic ecosystems. In combination with automated online FCM, they demonstrated a detection of contaminations based on alterations in the phenotypic fingerprint and thus α-diversity of the prokaryotic community *in situ* and in real-time. Establishing such approaches for microalgal cultures could contribute to contamination management or also support ecological engineering approaches. These insights could contribute to optimizing microalgal bioprocess feasibility by supporting the development of technological innovations for improved upstream performance. For instance, Haberkorn et al. (2021) showed that nanosecond pulsed electric field processing (nsPEF) could aid in fostering the upstream performance of microalgal feedstock production. Progress in implementing nsPEF on an industrial scale in non-axenic cultures has so far been hampered by a lack in understanding microalgal-bacterial interactions and the underlying intracellular treatment mechanisms. Automated online FCM combined with data analysis relying on phenotypic fingerprinting could perpetuate the understanding of the underlying microbial community responses. It provides real-time data on community diversity and insights into intrinsic cell responses following nsPEF treatments, by, for instance, depicting alterations in pigment, protein, and lipid content (Le Chevanton et al., 2013; Shurin et al., 2013; Cho et al., 2015; Zhang et al., 2018).

Automated online FCM is also a viable option for bioprocess optimization related to microalgal compound production. FCM is adaptable to myriad phenotypic assays and is, together with the automation module, compatible with various cultivation systems. Hence, it enables monitoring microalgal compounds, including proteins, lipids, or pigments *in situ* and at real-time (Hyka et al., 2013). Hence, automated online FCM could also aid in perpetuating microalgal production from monocultures through a quantification of microalgal counts in real-time, e.g., as response to external treatment stimuli. The assessment of compositional alterations in real-time, such as those related to pigment, lipid, or protein content, poses another option (Gao et al., 2020). The application of online FCM-based monitoring has yet been limited to the assessment of prokaryotes in aqueous ecosystems. Consequently, procedures and protocols for microalgal cultures are lacking. Thus, the study aimed to assess the feasibility of automated online FCM as an *in situ*, high-temporal resolution monitoring tool for the assessment of population dynamics in non-axenic *Chlorella vulgaris* cultures. The present study (1) provides a staining protocol and gating strategy that allow a simultaneous assessment (quantitative and diversity-related) of prokaryotes and microalgae in mixed ecosystems. It highlights the applicability harnessing industrially relevant phototrophic *C. vulgaris* in coculture with indigenous prokaryotes as the case study. (2) As a proof of concept, dynamic microbial events were tracked using *C. vulgaris* in five different ecosystems of defined and undefined cocultures with prokaryotes. (3) The study is the first to demonstrate the applicability of basic (detection and quantification) and advanced (phenotypic fingerprint) data analysis combined with automated online FCM to microalgal cultures.

## Materials and Methods

### Axenic *C. vulgaris* Culture

Axenic *C. vulgaris* SAG 211-12 was originally obtained from the culture collection of algae at Goettingen University, Germany. Cultures were maintained on modified diluted seawater nitrogen (DSN) medium agar plates (1.5% agar) using nitrate (141.65 g L^–1^ NaNO_3_) as nitrogen source under ambient conditions (30.3 μmol ⋅ photons ⋅ m^–2^ ⋅ s^–1^, 24 ± 1°C, ambient CO_2_ = 400 ppm) (Pohl et al., 1987; Haberkorn et al., 2020). For experiments, axenic cultures were grown by transferring single *C. vulgaris* colonies in 150-mL cultivation volume of sterile, modified DSN medium using 500-mL Erlenmeyer flasks. Cultures were stored in a shaking incubator (Multitron Pro; Infors AG, Bottmingen, Switzerland) applying cultivation conditions described by Haberkorn et al. (2019) for 7 days.

### Cocultures

*Tistrella mobilis* TH-33 (KF783213.1), *Pseudomonas pseudoalcaligenes* CLR9 (KF478199.1), and *Sphingopyxis* sp. AX-A (JQ418293.1) were maintained at −80°C in 80% vol/vol glycerol (80% vol/vol in dH_2_O). For experiments, all prokaryotic cultures were streaked out onto separate tryptic soy broth (TSB) agar plates (3% TSB, 1.5% agar) and incubated (30°C, 5 days). Subsequently, liquid cultures were prepared by transferring single bacterial colonies into 35 mL liquid TSB (3% in dH_2_O) and incubating at 30°C for 36 h.

For the experimental cultures, prokaryotic cells in the early exponential growth were used. Therefore, prokaryotic cell counts were quantified in the liquid cultures by manual FCM. Cultures were diluted with filtered (0.1-μm, Millex-GP, Millipore; Merck KGaA, Darmstadt, Germany) water (Evian; Danone, Paris, France) to a total cell concentration (TCC) below than 2.0 × 10^5^ cells ⋅ mL^–1^. Samples were stained with a SYBR^®^ Green I solution (working solution: 1:100 in 0.1-μm filtered dimethyl sulfoxide; Life Technologies, Eugene, OR, United States; final stain concentration: 1:10,000), incubated for 10 min at 37°C in the dark, and manually assessed on the flow cytometer and cell counts determined.

Based thereon, cultures were standardized to 10^7^ cells ⋅ mL^–1^ and washed three times in 35 mL modified DSN (10,000 × *g*; 5 min) to remove excess TSB. Subsequently, cultures were stored in 35-mL cultivation volume using sterile, modified DSN, and 100-mL Erlenmeyer flasks in a shaking incubator (Multitron Pro; Infors AG) at 30°C, 150 rpm, 70% relative humidity, 400 ppm CO_2_, and 36 μmol ⋅ photons ⋅ m^–2^ ⋅ s^–1^ until coculture establishment. Cocultures with *C. vulgaris* were established 16 h (*Tistrella* sp., *Sphingopyxis* sp.) or 4 h (*Pseudomonas* sp.) following standardization. See Table 1 for inoculation ratios of experimental cultures. Cocultures with three prokaryotic strains were established such that equal shares of each prokaryotic strain were obtained. The samples were cultivated in a shaking incubator (Multitron Pro; Infors AG) at 30°C, 150 rpm, 70% relative humidity, 400 ppm CO_2_, and 36 μmol ⋅ photons ⋅ m^–2^ ⋅ s^–1^ for 3 days. Technical constraints of the sampling robot allowed to assess one culture by automated online FCM. As the study did not encompass a fully ecological scope, but rather aimed at demonstrating the feasibility of automated online FCM for microalgae–prokaryote cocultures, each coculture experiment was conducted once.

**TABLE 1:** Coculture combinations of *C. vulgaris* with the prokaryotic strains *Sphingopyxis* sp., *Tistrella* sp., and *Pseudomonas* sp. assessed by automated online FCM, as well as corresponding initial and final cell concentrations [cells ⋅μL^–1^].

Coculture		Initial cell concentration [cells ⋅μL^–1^]		Final cell concentration [cells ⋅μL^–1^]	
		
		*C. vulgaris*	Prokaryotes	*C. vulgaris*	Prokaryotes
1	*C. vulgaris*–*Sphingopyxis* sp.	46.5	2.4	49.0	37.0
2	*C. vulgaris*–*Tistrella* sp.	27.2	2.2	48.0	28.4
3	*C. vulgaris*–*Sphingopyxis* sp., *Tistrella* sp., *Pseudomonas* sp. I	43.6	2.6	39.1	47.6
4	*C. vulgaris*–*Sphingopyxis* sp., *Tistrella* sp., *Pseudomonas* sp. II	66.4	126.6	66.7	1002.4
5	*C. vulgaris*–undefined; spontaneous, fortuitous contamination	45.6	2.2	49.2	39.9

*Cocultures were established and assessed as single cocultures (*n* = 1).*

### Flow Cytometry

All samples were measured on a BD Accuri^TM^ C6 Plus flow cytometer (BD Accuri Cytometers, San Jose, CA, United States) equipped with a 20-mW laser emitting at a wavelength of 488 nm. This allowed a collection of signals related to (1) FSC and (2) SSC light intensities, (3) green (533 ± 30 nm; FL1 channel), and (4) red fluorescence intensity (> 670 ± 25 nm; FL3 channel). The collection of those signals allowed to quantify (A) cell size, (B) cell granularity, (C) nucleic acid content (by SYBR^®^ Green I staining), and (D) chlorophyll autofluorescence, respectively. Before each experiment, the calibration of the flow cytometer was assessed with calibration beads (BD^TM^ CS&T RUO Beads; BD Biosciences, San Jose, CA, United States).

Manual flow cytometer measurements were always conducted with an analyzed volume of 50 μL, a flow rate of 66 μL ⋅ min^–1^, and a lower threshold of 800 on the FL1-H channel. Automated online FCM was conducted with a fully automated sampling, staining, and incubation robot (OC-300; onCyt Microbiology AG, Zurich, Switzerland) combined with the BD Accuri^TM^ C6 Plus flow cytometer. Samples were taken continuously at 25-min intervals throughout the entire experiment until termination on day 3 and measured using the same standard flow cytometer settings described for manual FCM. For each measurement point, a single sample was collected, diluted 1:100 with 0.1-μm filtered water (Evian; Danone), stained with SYBR^®^ Green I (working solution: 1:5,000 in 0.22-μm filtered 10 mM TRIS buffer, pH 8.0 containing 50 mM sodium thiosulfate; final stain concentration: 1:10,000) and incubated (10 min, 37°C). Subsequently, the sample was pumped to the flow cytometer and measured for 90 s (equivalent to an analyzed volume of approximately 61 μL). Between sampling, all internal tubing, the syringe pump, and the incubation/mixing chamber were rinsed with sodium hypochlorite solution (1% active chlorine), sodium thiosulfate solution (100 mM), and ultrapure water (Besmer et al., 2014, 2016, 2017a,b; Besmer and Hammes, 2016).

### Staining Protocol Validation

The operating principle of the staining robot for automated online FCM comprises first a sampling step from the culture, followed by an incubation with the stain, and subsequently a measurement step on the FCM, which was adopted for staining protocol validation. Additionally, the nucleic acid staining of prokaryotic communities harnessing SYBR^®^ Green I (37°C, 10 min) was shown to provide sensitive and reproducible quantitative data and phenotypic fingerprints on prokaryotes during automated online FCM (Besmer and Hammes, 2016; Besmer et al., 2016, 2017a,b; Props et al., 2018). As the study aimed at establishing a staining protocol for the simultaneous assessment of prokaryotes and microalgae, the feasibility of applying the staining protocol for the assessment of *C. vulgaris* was investigated. Six discreet subsamples of axenic *C. vulgaris* SAG 211-12 were stained with SYBR^®^ Green I. Each sample was measured manually and individually on the flow cytometer in quintuplicates applying the same conditions as described in section “Flow Cytometry” assessing cells at a staining temperature of 37°C for 5, 8, 10, and 15 min. Additionally, the effect of the staining temperature was investigated by staining cells for 10 min at 4, 37, and 40°C. Negative controls were analyzed using SYBR^®^ Green I in filtered water only, following the same staining protocol.

### Gating

**FIGURE 1:**
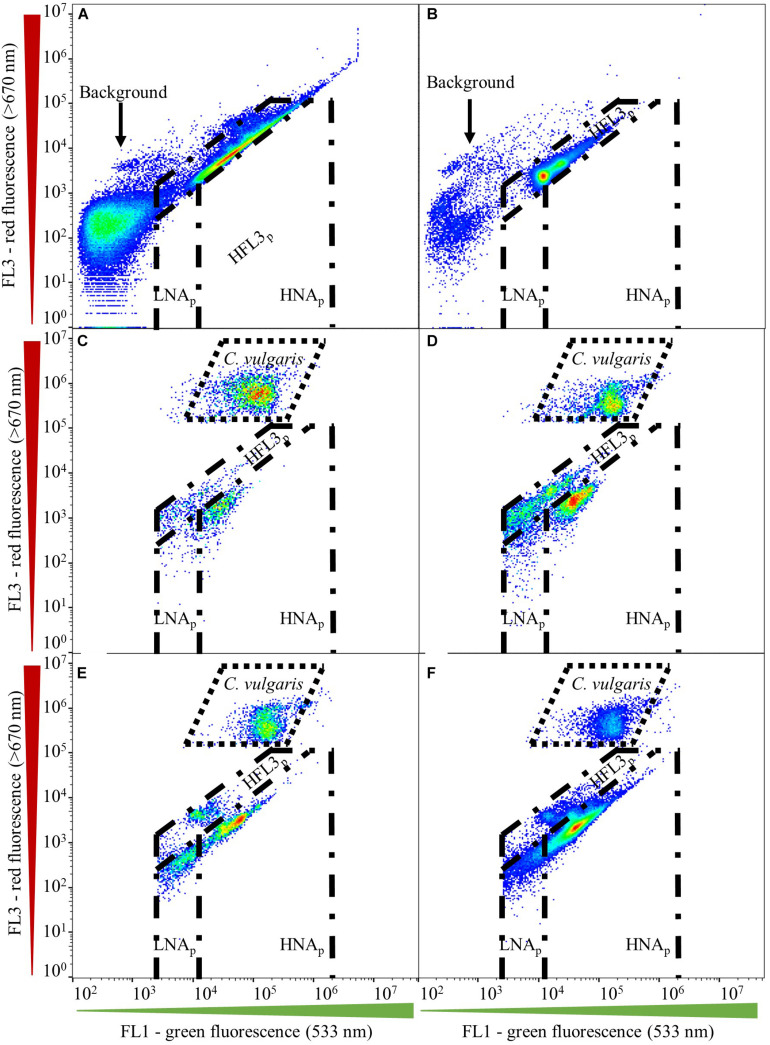
Gating strategy established using dual-density-plots on the green (FL1) and red (FL3) fluorescent channels. Gates, including microalgal, HNA_p_, LNA_p_, and HFL3_p_ domains, are shown for **(A)** axenic *Tistrella* sp., **(B)** axenic *Sphingpyxis* sp., **(C)** *Tistrella* sp.–*C. vulgaris* coculture, **(D)** *Sphingopyxis* sp.–*C. vulgaris* coculture, and *Sphingpyxis* sp., *Tistrella* sp., *Pseudomonas* sp.–*C. vulgaris* coculture inoculated to a lower **(E)** and higher **(F)** initial prokaryotic concentration than *C. vulgaris*.

Gate establishment for microalgae first encompassed assessing fresh and axenic *C. vulgaris* culture. Aliquots of the same axenic *C. vulgaris* sample were diluted with filtered (0.1-μm, Millex-GP, Millipore; Merck KGaA) water (Evian; Danone) to obtain a cell concentration below 2.0 × 10^5^ cells ⋅ mL^–1^. Subsequently, six discrete sub-samples were stained (37°C, 10 min) with SYBR^®^ Green I (working solution: 1:5,000 in 0.22-μm filtered 10 mM TRIS buffer, pH 8.0 containing 50 mM sodium thiosulfate; final stain concentration: 1:10,000) in the dark and measured in quintuplicate (Besmer et al., 2017b). Samples were measured manually and separately on the flow cytometer. Negative controls were analyzed using SYBR^®^ Green I in filtered water only, following the same staining protocol. The microalgal gate was established based on green (FL1) and red (FL3) fluorescent intensities. Microalgal gates were validated for their fit throughout all samples obtained in this study (Figures 1C–F). Gates for assessing prokaryotes in coculture with *C. vulgaris* were initially adopted from Prest et al. (2013) and included prokaryotic regions for low (LNA_p_) and high nucleic acid content (HNA_p_) organisms (Figures 1A–F). Prokaryotic gates were validated for their fit throughout all samples by first assessing axenic prokaryotic cultures, followed by applying the gates to prokaryotes in coculture with *C. vulgaris*. No compensation was applied.

### Data Analysis

Raw data were collected with the BD Accuri^TM^ C6 software (v1.0.1; BD Accuri Cytometers). Each measurement point generated a single FCS file, which was exported to the R statistical environment (R-Studio, v1.1.456). Data and statistical assessment were performed using the functionalities offered by the *flowCore* (v1.38.2) and *Phenoflow* (v1.1.2) packages. Virtual gating was applied following the gating strategy described in *Staining Protocol Validation*.

For data obtained from automated online FCM of the established cocultures, basic FCM data analysis (detection and quantification) was conducted for both *C. vulgaris* and prokaryotic (HNA_p_ gate) communities. Data analyses relying on phenotypic fingerprints, and based thereon the phenotypic diversity index, were established for the prokaryotic community employing the HNA_p_ domain of the multispecies assemblages and the undefined culture to assess shifts in the prokaryotic community relating to phenotypic diversity-based alterations. Therefore, the *Diversity_rf* function was used (number of bootstraps, *n* = 3), employing an adapted analytics approach initially suggested by Props et al. (2018) for prokaryotes in aquatic ecosystems. Briefly, the function performs bivariate kernel density estimation on selected phenotypic traits (FL1-A, FL3-A, FSC-A, and SSC-A) and concatenates the obtained values to a one-dimensional feature vector, the phenotypic fingerprint. The phenotypic fingerprint then serves for calculating the phenotypic diversity index. In analogy to taxonomy, i.e., relative abundance-based α-diversity, the phenotypic diversity index resembles the “effective number of phenotypic states” in a microbial community. Following Props et al. (2018), the Hill-diversity metric of order two was employed as α-diversity measure to put equal weight on the richness and evenness.

A *t* test was performed to statistically assess data obtained from gate establishment. A non-significant Shapiro–Wilk test (*P* > 0.05) and *F* test (*P* > 0.05) indicated normal distribution and homogeneity of variances of the obtained data, respectively. A Wilcoxon rank-sum test was conducted to assess statistical significances of data collected from staining protocol validation, as the data were not normally distributed.

## Results

### Gate Definition

Applying the microalgal gate based on the emission spectra of SYBR^®^ Green I and of chlorophyll (autofluorescence) provided a valid and reproducible approach to assess microalgal cell counts in fresh *C. vulgaris* samples. No significant difference was found between the datasets collected on the FSC/SSC and FL1/FL3 channels (*t* test; *t* = -2.09, *df* = 10; *P* = 0.06; *n* = 30). Quantitative assessment revealed a relative standard deviation of 3.1% and 3.2% of counts collected on the FSC/SSC and FL1/FL3 channels, respectively, indicating low intrasample variation. Counts collected on the FL1/FL3 channels were shown to represent *C. vulgaris* biomass yields, indicating that the staining protocol (SYBR^®^ Green I; 37°C, 10 min) and subsequent count determination employed in this study represented *C. vulgaris* cell counts and biomass yields well (Haberkorn et al., 2019). No shift was observed in microalgal nucleic acid or chlorophyll content throughout the cocultures assessed, which was indicated by a 100% coverage within the gate established on the FL1/FL3 fluorescent channels.

In the prokaryotic domain, the emission collected on the FL1/FL3 fluorescent channels revealed the presence of different clusters (Figures 1A–F). Gates for assessing prokaryotic populations were initially adopted from Prest et al. (2013), who proposed a discrimination of prokaryotic regions characterized by low (LNA) and high nucleic acid (HNA) content. Albeit axenic prokaryotic cultures located in the HNA domain suggested by Prest et al. (2013), coculture with *C. vulgaris* resulted in a shift of the localization of prokaryotes on the FL1/FL3 channels toward lower emission on the green (FL1) and red (FL3) fluorescent channels (Figures 1A–D). Additionally, prokaryotes in the multispecies assemblage located at the intersection of the initial LNA and HNA domains proposed by Prest et al. (2013) prevented a clear discrimination of the two populations (Figures 1E,F). Hence, LNA_p_ and HNA_p_ gates for assessing prokaryotes in coculture with *C. vulgaris* required adaptation toward lower emission on the green (FL1) (1.5 × 10^4^) and red (FL3) fluorescent spectrum. For a majority of cocultures, counts collected in the LNA_p_ gate were negligible and might have been associated with background scattering.

Cocultures, such as those established with *Sphingopyxis* sp. (Figure 1D) and three prokaryotic strains (Figures 1E,F), indicated the presence of an additional prokaryotic cluster that emitted higher on the red fluorescence (FL3) channel, resulting in the establishment of a third gate denoted as HFL3_p_. However, signal collected within the HFL3_p_ gate could not be confirmed for all cocultures. In fact, counts obtained within the HFL3_p_ gate could quantitatively negligible during coculture. Some studies describe those signals collected in the HFL3_p_ domain to be associated with background noise or scattering (Hammes et al., 2008; Hammes and Egli, 2010; Prest et al., 2013). In addition, the presence of a HFL3_p_ domain yet remains unreported for microalgal and prokaryotic aquatic ecosystems. Hence, diversity assessment of prokaryotic communities including HFL3p fractions would be speculative. Thus, the HFL3p fraction was excluded from subsequent community diversity analysis.

Indistinct signal at fluorescent intensities lower than those proposed for the LNA_p_ domain on the green (FL1) fluorescent channel, as well as at higher fluorescent intensities on the red (FL3) fluorescent channel, was associated with background scattering and thus excluded from further analysis (Figures 1A,B; Berney et al., 2008).

### Staining Protocol Validation

**FIGURE 2:**
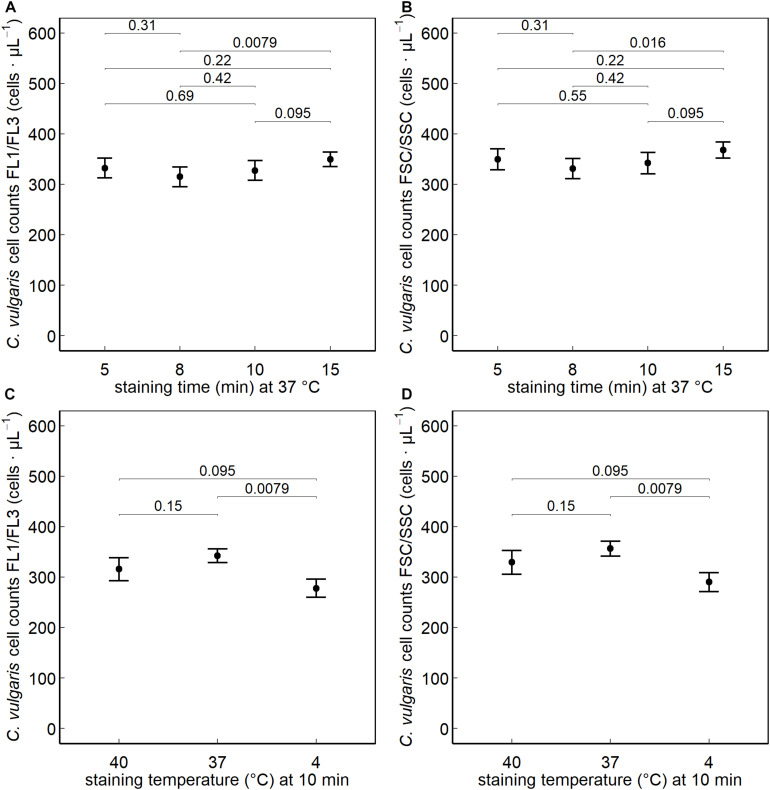
*Chlorella vulgaris* cell counts collected on the green (FL1) and red (FL3) fluorescent and FSC/SSC channels after nucleic acid staining with SYBR^®^ Green I. The effect of varying the staining times (min) at 5, 8, 10, and 15 min at a staining temperature of 37°C **(A,B)** and the effect of the different staining temperatures 40°C, 37°C, and 4°C at a staining time of 10 min **(C,D)** were assessed (*n = 30*).

No significant difference was observed between a staining time of 10, 5, 8, or 15 min (at 37°C) on the FL1/FL3 fluorescent or on the FSC/SSC channels. Significantly higher (*P* < 0.05) counts were obtained when staining cells at 37°C (10 min) than at 4°C, whereas no difference was observed when increasing the staining temperature to 40°C (Figure 2).

### Online Monitoring of Culture Dynamics

Establishing gates for prokaryotes and *C. vulgaris* based on the intensities collected on the green (FL1) and red (FL3) fluorescent channels enabled the discrimination of microalgal and prokaryotic populations and thus a simultaneous quantitative and diversity-related (multispecies assemblage, undefined coculture) assessment during automated online FCM.

**FIGURE 3:**
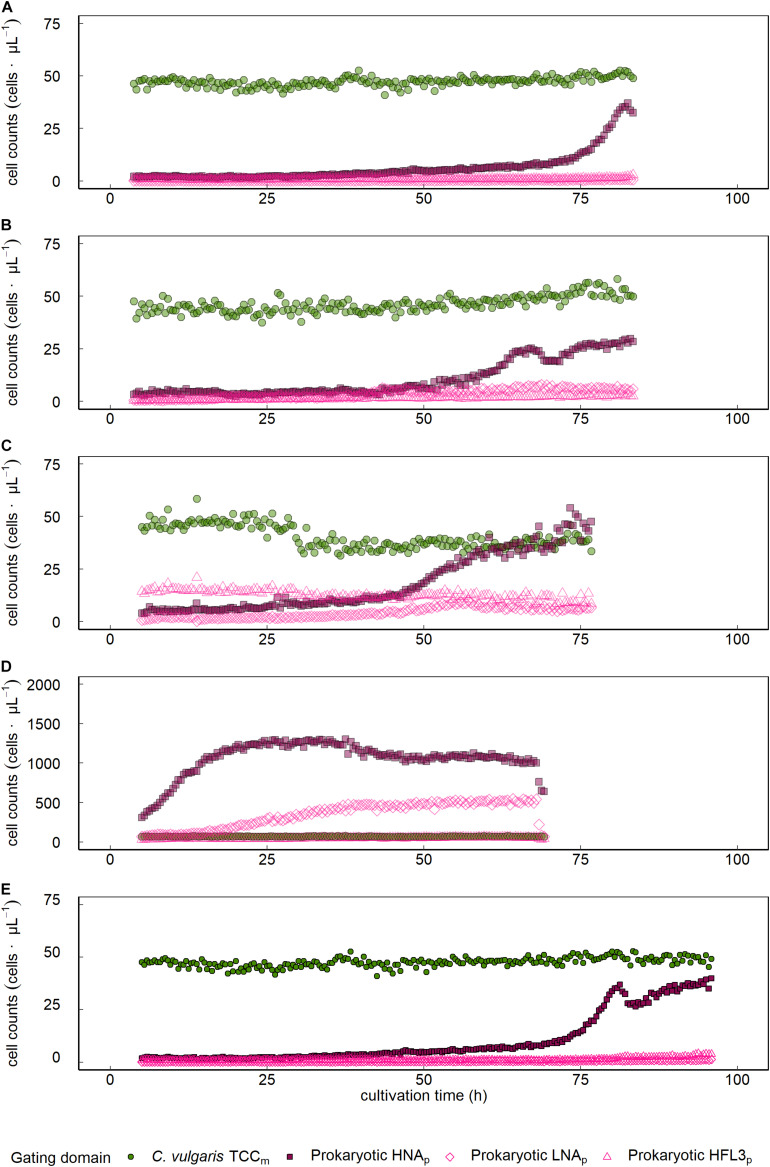
Growth dynamics of *C. vulgaris* and prokaryotes in mixed ecosystems. Data is presented for single cocultures that were established from *C. vulgaris* with **(A)** *Sphinogpyxis* sp., **(B)** *Tistrella* sp., *Sphinogpyxis* sp., *Tistrella* sp., and *Pseudomonas* sp. inoculated to a **(C)** lower or **(D)** higher initial concentration than *C. vulgaris*, and **(E)** a coculture based on spontaneous, fortuitous contamination of axenic *C. vulgaris* cultures. FCM assessed *C. vulgaris* total cell concentration (TCC_m_).

*Sphingopyxis* sp. showed a 15.1-fold count increase from 2.4 to 37.0 cells ⋅μL^–1^ with an initial lag phase lasting the first 72.5 h of cultivation and subsequent exponential growth phase, not outnumbering *C. vulgaris* throughout the entire cultivation period (Figure 3A and Table 1). Visual inspection of FL1/FL3 fluorescent intensities indicated the presence of a prokaryotic cluster that was emitting higher on the red (FL3) fluorescent channel (Figure 1D). *Tistrella* sp. counts increased 13.1-fold during cultivation but did not surpass *C. vulgaris* cell counts (Figure 3B and Table 1). An initial lag phase lasted approximately 50 h followed by an accelerated growth phase until the end of the cultivation period. Most of the counts were collected in the HNA_p_ domain. With continuing cultivation, the share of cells located in the HFL3_p_ gate increased, leading to a maximum of 8 cells ⋅μL^–1^. However, visual inspection of density plots obtained on the FL1/FL3 fluorescent channels did not show distinct clusters or patterns that would substantiate the presence of *Tistrella* sp. in the HFL3_p_ gate.

**FIGURE 4:**
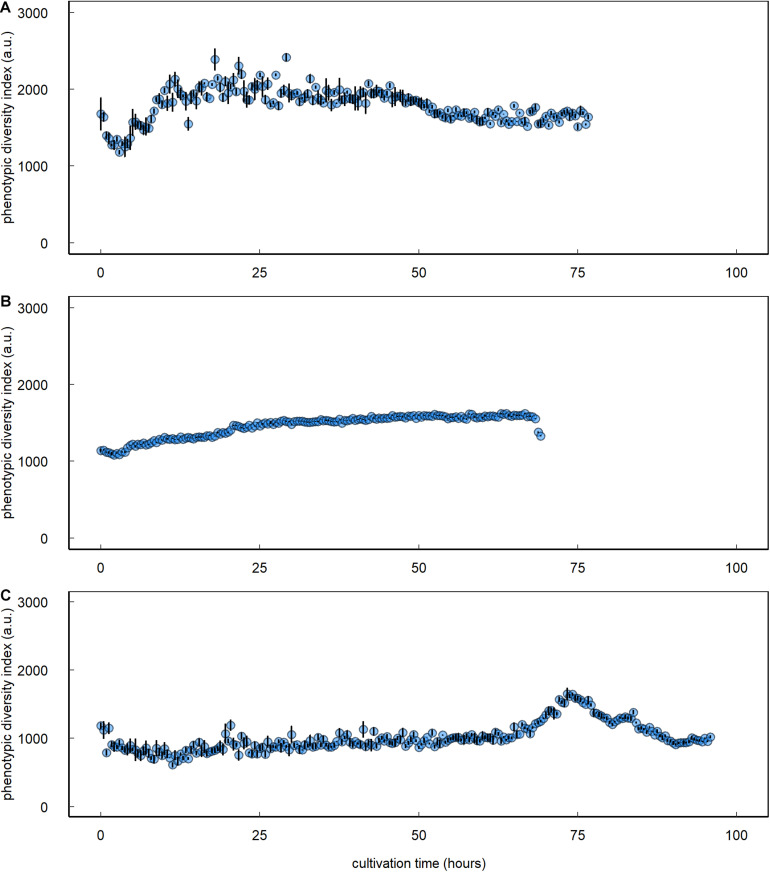
Prokaryotic phenotypic diversity index of *C. vulgaris*–prokaryote mixed ecosystems. Data are presented for single cocultures that were established from *C. vulgaris* with *Sphingopyxis* sp., *Tistrella* sp., and *Pseudomonas* sp. inoculated to **(A)** a lower or **(B)** higher initial concentration than *C. vulgaris* and **(C)** a coculture based on spontaneous, fortuitous contamination of axenic *C. vulgaris* cultures. Error bars denote bootstrap errors (*n* = 3).

Inoculating the multispecies assemblage of *C. vulgaris* and *Tistrella* sp., *Sphingopyxis* sp., and *Pseudomonas* sp. to a lower concentration than *C. vulgaris* resulted in an 18.7-fold prokaryotic count increase with an initial lag phase followed by an exponential growth phase that started approximately 50 h after inoculation (Figure 3C and Table 1). During the initial lag phase, clusters within the HFL3_p_ gating domain were observed, which remained at constant 12.8 ± 2.2 cells ⋅μL^–1^ throughout the entire cultivation period. During culture of the multispecies assemblage, the phenotypic diversity index increased by 59.7% from initial 1,465.5 ± 89.1 a.u. to a maximum of 2,341.1 ± 66.6 a.u. approximately 26 h after inoculation followed by a decline of 28.2% to 1,680.5 ± 19.3 a.u. at the end of the cultivation period (Figure 4A). Inoculating the multispecies assemblage to a higher concentration than *C. vulgaris* resulted in an immediate incidence of exponential prokaryotic growth for the first 37.5 h of cultivation, with a clear dominance of counts collected in the HNA_p_ gating domain (Figure 3D and Table 1). Although the maximum observed prokaryotic cell concentration amounted to 1,305.7 cells ⋅μL^–1^ (37.5 h after inoculation), the overall prokaryotic count increase was only 10-fold. After reaching a maximum of 1,305.7 cells ⋅μL^–1^, prokaryotic counts decreased 1,002.4 cells ⋅μL^–1^. Cells collected in the HFL3_p_ gating domain were high at a constant concentration of 47.6 ± 9.2 cells ⋅μL^–1^ throughout the cultivation period. The phenotypic diversity index increased by approximately 17.1% from initial 1,131.4 ± 21.7 a.u. to a maximum of 1,324.5 ± 6.0 a.u. (Figure 4B).

The protocol was also applicable to prokaryotes in the coculture based on short-term spontaneous fortuitous contamination. Prokaryotes initially showed decelerated growth, followed by an exponential increase at the end of the cultivation period leading to a maximum cell concentration of 39.9 cells ⋅μL^–1^, where the HNA_p_ fraction clearly dominated (Figure 3E and Table 1). The phenotypic diversity index initially showed no decisive pattern fluctuating between 2,000 and 3,000 a.u. but revealed a distinct peak with a maximum of 1,553 ± 39.0 a.u. 78.4 h after inoculation followed by a decline to 944.1 ± 10.2 a.u. at the end of the cultivation period (Figure 4C).

## Discussion

Because microalgae have emerged as a next-generation biotechnological production system for the biobased domain, delivering feedstock and high-value components, the economization of their connected value chains remains a main target. Important for optimizing the reproducibility and productivity of microalgal feedstock production are stable cultures, which can be supported by the *in situ*, real-time monitoring and management of culture ecologies. The study showed that the protocol employed harnessing chlorophyll autofluorescence and nucleic acid staining based on SYBR^®^ Green I in conjunction with automated online FCM provided a rapid and sensitive approach for microalgal culture assessment.

A clear advantage of microalgae is their naturally occurring pigments and size, which allows distinguishing them from abiotic particles, other microalgal species, or bacteria. In this study, harnessing the autofluorescence of chlorophyll enabled discriminating *C. vulgaris* from bacteria and abiotic particles using the red fluorescent (FL3, > 670 nm) channel and thus facilitated gating microalgae during coculture. Microalgal pigments, including chlorophyll, are gaining increasing relevance as bioproducts for the industrial exploitation of microalgae. Chlorophyll, for example, has applications in the cosmetics, food, pharmaceutical, and nutraceutical domain (da Silva Ferreira and Sant’Anna, 2017). The feasibility of microalgal bioproduct, i.e., pigment assessment harnessing their naturally occurring autofluorescence, opens promising applications. Automated online FCM could be employed as an online and inline monitoring and management tool for optimizing bioprocesses associated with microalgal pigment production. Industrially relevant microalgae, such as *Dunalliella salina*, *Haematococcus pluvialis*, and *Scenedesmus almeriensis*, which lack in chlorophyll, are producers of other commercially exploited, high-value pigments, such as carotenoids including astaxanthin (excitation: 488 nm; emission: 675 nm) (Ukibe et al., 2008; Enzing et al., 2014). Harnessing the autofluorescence of those pigments by FCM allows circumventing chemical or toxic staining, extraction, and analysis protocols, whereas automated online FCM provides the additional advantage of real-time data acquisition for bioprocess management.

Microalgal pigment content does not necessarily correlate with biomass yields but responds to variations in light, temperature, and nutrient availability, which impedes its use as a measure for biomass yield quantification (da Silva Ferreira and Sant’Anna, 2017). To enable a quantitative assessment of cocultures, cells were stained with SYBR^®^ Green I (37°C, 10 min), which was shown to enable a sensitive quantification of prokaryotes by FCM with limits at cell concentrations as low as 0.1–1 cells ⋅μL^–1^ (Prest et al., 2013; Besmer and Hammes, 2016; Besmer et al.,2017a,b). In this study, no significant difference was observed between *C. vulgaris* counts collected on the FSC/SSC and FL1/FL3 fluorescent channels. Hence, the nucleic acid staining protocol employed (SYBR^®^ Green I, 37°C, 10 min) served as a valid and reproducible approach for quantifying microalgal and prokaryotic counts simultaneously during coculture by automated online FCM. Accordingly, Haberkorn et al. (2019) showed that axenic *C. vulgaris* counts collected on the FL1/FL3 channels following a staining with SYBR^®^ Green I corresponded well with those collected on the FSC/SSC channels and actual *C. vulgaris* biomass yields.

The staining protocol employed enabled a sensitive, rapid quantification of microalgal and prokaryotic populations at different concentrations and of different complexity. Automated online FCM enabled discriminating different growth phases of prokaryotes, as well as fluctuations and concentration peaks at high-temporal resolution and within a broad concentration range (2.2–1,002.4 cells ⋅μL^–1^). Prokaryotic growth in coculture was characterized by lag phases lasting up to or longer than 2 days, while other cultures showed an immediate incidence of exponential growth. Individual cocultures and the multispecies assemblage inoculated to a lower concentration than *C. vulgaris* yielded higher prokaryotic counts than the multispecies assemblage that was inoculated to a higher concentration than *C. vulgaris*. Inoculating prokaryotes to a higher concentration than *C. vulgaris* resulted in an immediate incidence of exponential prokaryotic growth followed by an 8-fold prokaryotic count increase. Conversely, inoculating prokaryotes to a lower concentration than *C. vulgaris* resulted in an extended lag phase and an 18.7-fold prokaryotic count increase. Oligotrophic environments were reported as being dominated by slow-growing prokaryotic populations (Klappenbach et al., 2000). The high salt content and the absence of organic carbon sources in the initial DSN medium suggest a classification of the environment as oligotrophic promoting slow-growing prokaryotes. Both bacteria and microalgae were shown as being capable of releasing dissolved organic carbon into the environment in coculture providing a carbon source for growth, which might have supported prokaryotic growth even under oligotrophic conditions (Cho et al., 2015). The ability of automated online FCM in conjunction with the established staining protocol (SYBR^®^ Green I, 37°C, 10 min) to depict prokaryotic community dynamics *in situ*, in real-time, and at high-temporal resolution covering different concentration ranges yields promising applications of the technology as an online and inline monitoring tool during microalgal culture. Hence, incorporating automated online FCM into microalgal feedstock production could support culture management, as it enables taking immediate countermeasures in case of contamination or prokaryotic upsurge.

In the prokaryotic gating domain, the emissions collected on the FL1/FL3 fluorescent channels revealed the presence of different clusters. SYBR^®^ Green I is sensitive toward nucleic acids, including DNA and RNA (Proctor et al., 2018). Assuming a complete penetration of the stain into the cells, shifts in the green (FL1) fluorescence intensity can occur because of alterations in the nucleic acid content, for instance, that observed for different prokaryotic communities owing to their difference in genome size or during different growth stages of prokaryotes (Prest et al., 2013; Buysschaert et al., 2018). Various studies have described in depth and also characterized prokaryotic HNA and LNA fractions in aquatic ecosystems, resulting in a gating approach as suggested by Prest et al. (2013) (Lebaron et al., 2002; Bouvier et al., 2007; Besmer et al., 2014, 2017a; Props et al., 2018; Zhao et al., 2018). Initial gate establishment for prokaryotic communities encompassed adopting those gates suggested by Prest et al. (2013) to enable discriminating the different clusters. However, employing the same gating strategy did not allow for a clear discrimination of HNA and LNA prokaryotic fractions on the FL1/FL3 fluorescent channels. Instead, prokaryotes showed lower emission on the green fluorescence channel (FL1) in coculture with *C. vulgaris* and thus located at the fringe of the suggested border separating HNA and LNA domains demanding an adaptation tailored to the *C. vulgaris* cocultures investigated in this study. HNA and LNA fractions appear as two domains separated by their fluorescence intensity on the green fluorescent channel (FL1) after staining with SYBR^®^ Green I. The required shift of the gating domains might relate to a deceleration of prokaryotic metabolic activity in coculture with *C. vulgaris* and consequently lower fluorescence intensity on the green fluorescence (FL1) channel. Hyka et al. (2013) reported that the nucleic acid content of microalgae can also fluctuate, depending on the phase of the cell cycle, but no alterations were observed in this study. Additionally, prokaryotic presence or elevated growth did not affect the nucleic acid content of *C. vulgaris*, which was indicated by 100% coverage in the established gate and the applicability of the same gate to *C. vulgaris* in axenic and non-axenic cultures. However, future studies employing automated online FCM for assessing microalgal dynamics over longer cultivation periods or the impact of processing on microalgal physiology might consider adapting the proposed gate toward higher or lower emission on the green fluorescent (FL1) channel. Most prokaryotic counts were collected in the established HNA_p_ gating domain. LNA_p_ fractions, on the other hand, were characterized by low counts. These observations are in accordance with other studies showing that the majority of read counts in aquatic, prokaryotic ecosystems are associated with the HNA domain (Prest et al., 2013; Besmer et al., 2016; Proctor et al., 2018). In turn, the presence of prokaryotes characterized by LNA contents was confirmed by several studies for aquatic ecosystems. However, LNA_p_ domains yet remain unreported for microalgal ecosystems. Although the presence of LNA content prokaryotes in this study cannot be excluded, a clear identification of LNA prokaryotes remains challenging for two reasons. First, the LNA_p_ fraction was characterized by low counts. The corresponding gating domain might have also captured counts from background scattering. Hence, it remains questionable whether the counts captured in the LNA_p_ domain might have been affiliated with background noise or LNA-content prokaryotes. Second, a partially unclear allocation of LNA_p_ counts, i.e., a location at the fringe of the gating border, to the gating domain further impeded a clear identification of prokaryotic clusters that might have been associated with an LNA_p_ domain. An unclear allocation of counts into the gating domain supports the assumption that counts collected in the LNA_p_ gating domain were related to background scattering. Furthermore, an unclear allocation could relate to changes in the metabolic state of cells that lead to a shift in their location on the density plots obtained from FCM. For instance, bacterial sporulation, such as that reported for species of the order *Bacillales*, increases the level of dye uptake, resulting in higher emission on the respective channel (Zhang et al., 2020). Members affiliated with the order of *Bacillales* were also reported for microalgal cultures (Steichen et al., 2020). To study those interactions, fluorescence-activated cell sorting would allow separating the different prokaryotic populations of interest. Combining the sorting with taxonomic assessments based on, for instance, 16S rDNA amplicon sequencing would allow identifying the populations of interest. Subsequently, more complex interactions between selected prokaryotic species of each fraction with microalgae could be studied employing engineered cocultures.

Hence, an LNA_p_ domain as suggested by Prest et al. (2013) was not applicable in the cocultures assessed in this study and was thus excluded from further diversity analysis. However, future studies investigating more complex microalgal ecosystems might relate back to an LNA_p_ gating domain for a prokaryotic diversity assessment or contamination monitoring, as several studies have shown the existence of prokaryotes in complex aquatic ecosystems to locate in the LNA_p_ domain (Proctor et al., 2018). Cocultures, such as those established with *Sphingopyxis* sp. (Figure 1D) and three prokaryotic strains (Figures 1E,F), indicated the presence of an additional prokaryotic cluster that emitted higher on the red fluorescence (FL3) channel, which was denoted as HFL3_p_ domain. Interestingly, higher emittance on the red fluorescence (FL3) channel was observed for prokaryotes, including *Sphingopyxis* sp. in individual coculture with *C. vulgaris*, as well as in both multispecies assemblages. Certain members affiliated with *Sphigomonadaceae* were reported being capable of pigment formation, involving carotenoids, such as asthaxanthin or bacteriochloropohyll a, which can cause an increase of the red fluorescence (FL3) intensity (Rosenberg et al., 2014). The rise-time periods of the HFL3_p_ fraction observed during those cocultures could thus relate to an induction of pigment formation or to a count increase of cells forming those pigments during coculture. However, the presence of prokaryotes located in the HFL3_p_ domain could not be confirmed for all cocultures. In fact, counts obtained within the HFL3_p_ were quantitatively negligible during coculture. Some studies describe those signals collected in the HFL3_p_ domain to be associated with background noise or scattering (Hammes et al., 2008; Hammes and Egli, 2010; Prest et al., 2013). Although low counts might not serve as sole exclusion criterion of the HFL3_p_ cluster, a lack in stable occurrence throughout all cocultures combined with an open affiliation of the cluster to prokaryotic organisms and the resultant potential of bias through background noise inclusion led to the exemption of the HFL3_p_ cluster from the diversity analysis in this study.

Combining automated online FCM with data analysis relying on phenotypic fingerprinting based on inherent cell characteristics provides a powerful tool for detecting, tracking, and quantifying prokaryotic disturbances or contaminations and could also pose a viable option for microalgal cultures (Buysschaert et al., 2018; Props et al., 2018). In this study, diversity assessment based on prokaryotic phenotypic fingerprints did not allow for a discrimination of different prokaryotic strains, which could relate to similarities in the phenotypic parameters assessed. But phenotypic fingerprinting indicated that the differences in prokaryotic growth patterns were associated with a dominance of one or two strains within the multispecies assemblage. For the multispecies assemblage with prokaryotes inoculated to a lower concentration than *C. vulgaris*, the phenotypic diversity index increased 1.6-fold within the initial 26 h of cultivation. Conversely, the phenotypic diversity index only gradually increased for the multispecies assemblage with higher initial prokaryotic counts leading to a maximum 1.2-fold increase at the end of the cultivation period. An increase in the phenotypic diversity index relates to an increase in the evenness component and thus equalization of the different community members (Props et al., 2018). This equalization could relate to an assimilation of phenotypic characteristics among community members. Hence, phenotypic fingerprinting indicated that during coculture of the multispecies assemblage inoculated to higher prokaryotic than *C. vulgaris* concentration, one or two of the three strains dominated throughout the entire cultivation period that governed the growth performance and resulted in overall decelerated prokaryotic growth. The information obtained by automated online FCM, combined with data analysis relying on phenotypic fingerprinting, poses a powerful tool that could improve not only the understanding of population dynamics underlying complex ecosystems but also their response to external events. For instance, the implementation of emerging processing technologies, such as nsPEF in single cell–based biorefineries, remains hampered by a lack of understanding the underlying ecosystem responses or treatment mechanisms (Buchmann and Mathys, 2019; Haberkorn et al., 2021). This situation could be overcome by implementing automated online FCM in combination with data analysis approaches relying on phenotypic fingerprinting to assess responses in real-time. Additionally, Helisch et al. (2020) highlight the importance of long-term stability of non-axenic microalgae-based ecosystems as crucial to establish life-support systems for long-term space exploration, which demands *in situ* monitoring tools, such as automated online FCM, which provide data at high-temporal resolution for optimal process control.

## Conclusion

Automated online FCM poses a powerful technology for improving the feasibility of microalgal feedstock production through providing data on culture dynamics *in situ* and at high-temporal resolution. Harnessing emissions collected on the FL1/FL3 fluorescent channels, obtained by nucleic acid staining and chlorophyll autofluorescence, enables a simultaneous assessment of prokaryotes and *C. vulgaris* in artificially engineered and natural cultures over a broad concentration range (2–1,002 cells ⋅μL^–1^). Automated online FCM in combination with data analysis relying on phenotypic fingerprinting provides information on quantitative and diversity-related community dynamics. Simultaneously, the study highlights different prokaryotic community fractions in microalgal cultures. Differences in the nucleic acid content and pigmentation could allow distinguishing them by FCM. In that context, characterizing non-axenic *C. vulgaris* cultures beyond phenotypic assessments proposed in this study on a taxonomic base could further advance automated online FCM by identifying populations of interest. Such assessments provide a better understanding of the underlying microbial network interactions. The study lays the foundations for an application of automated online FCM implying far-reaching applications to impel and facilitate the implementation of innovations targeting at microalgal bioprocesses optimization.

## Data Availability Statement

The raw data supporting the conclusions of this article will be made available by the authors, without undue reservation.

## Author Contributions

IH and CO: data acquisition. IH: manuscript drafting. LB, MB, and AM: final approval of the manuscript. All authors: conceptualization, study design, technical support, analysis, data interpretation, and critical revision.

## Conflict of Interest

LB was employed by company Bühler AG. MB was employed by onCyt Microbiology AG. The remaining authors declare that the research was conducted in the absence of any commercial or financial relationships that could be construed as a potential conflict of interest.

## Abbreviations

FCM: flow cytometry
FSC: forward scattered light intensities
HFL3: higher red fluorescence emission
HNA: high nucleic acid
LNA: low nucleic acid
SSC: sideward scattered light intensities
TCC: total cell concentration.
